# Marine Robotics for Deep-Sea Specimen Collection: A Taxonomy of Underwater Manipulative Actions

**DOI:** 10.3390/s22041471

**Published:** 2022-02-14

**Authors:** Angela Mazzeo, Jacopo Aguzzi, Marcello Calisti, Simonepietro Canese, Michela Angiolillo, A. Louise Allcock, Fabrizio Vecchi, Sergio Stefanni, Marco Controzzi

**Affiliations:** 1 The BioRobotics Institute, Scuola Superiore Sant’Anna, 56127 Pisa, Italy; marco.controzzi@santannapisa.it; 2 Department of Excellence in Robotics & AI, Scuola Superiore Sant’Anna, 56127 Pisa, Italy; 3 Stazione Zoologica Anton Dohrn (SZN), 80121 Naples, Italy; jaguzzi@icm.csic.es (J.A.); simonepietro.canese@szn.it (S.C.); michela.angiolillo@isprambiente.it (M.A.); fabrizio.vecchi@szn.it (F.V.); sergio.stefanni@szn.it (S.S.); 4 Instituto de Ciencias del Mar (ICM)—Consejo Superior de Investigaciones Científicas (CSIC), 08003 Barcelona, Spain; 5 Lincoln Institute for Agri-Food Technology, University of Lincoln, Lincoln LN6 7TS, UK; mcalisti@lincoln.ac.uk; 6 Istituto Superiore per la Protezione e la Ricerca Ambientale (ISPRA), 00144 Rome, Italy; 7 Ryan Institute and School of Natural Sciences, NUI Galway, H91 TK33 Galway, Ireland; louise.allcock@nuigalway.ie

**Keywords:** underwater gripper, ROV gripper, underwater manipulation, underwater end-effector, robotic underwater hands, marine biological sampling, taxonomy of actions

## Abstract

In order to develop a gripping system or control strategy that improves scientific sampling procedures, knowledge of the process and the consequent definition of requirements is fundamental. Nevertheless, factors influencing sampling procedures have not been extensively described, and selected strategies mostly depend on pilots’ and researchers’ experience. We interviewed 17 researchers and remotely operated vehicle (ROV) technical operators, through a formal questionnaire or in-person interviews, to collect evidence of sampling procedures based on their direct field experience. We methodologically analyzed sampling procedures to extract single basic actions (called atomic manipulations). Available equipment, environment and species-specific features strongly influenced the manipulative choices. We identified a list of functional and technical requirements for the development of novel end-effectors for marine sampling. Our results indicate that the unstructured and highly variable deep-sea environment requires a versatile system, capable of robust interactions with hard surfaces such as pushing or scraping, precise tuning of gripping force for tasks such as pulling delicate organisms away from hard and soft substrates, and rigid holding, as well as a mechanism for rapidly switching among external tools.

## 1. Introduction

The versatility of the human hand in manipulation is still barely emulated by robotic grippers [1,2]. In the last few decades, industrial production line needs have been effectively satisfied by single-purpose end-effectors that can be programmed to perform repetitive specific tasks at high speeds [3]. Some other application areas for robotic grippers (i.e., prosthetics, collaborative and service robotics or manipulation in unstructured environments) require a higher degree of adaptability in order to handle the variability of the manipulated objects or the purpose of the action. Coherently, industry 4.0 is pushing toward flexibility and reconfigurability in robotic design and manipulation task planning [4,5].

If robotic manipulation is demanding on land, issues become even more evident in challenging environments such as space, oceanic depths, and hazardous or disaster sites. Bringing human manipulation capabilities to harsh environments is a challenge that must overcome traditional design and robotic concepts [2], and presents a multidisciplinary problem. It involves, for example, a choice of design materials that can withstand particular conditions, additional dynamic components that must be accounted for in control architecture, sensory redundancy to counteract the occurrence of any occlusion (e.g., [6]), management of communication issues, and so on. In the case of teleoperated systems, manipulation possibilities depend also on embodiment, control algorithms, proper human–machine interface, and the teleoperator experience; in the case of autonomous manipulation, they depend on the way decision capabilities are implemented in a manipulation task planner.

### 1.1. Human Hand Manipulation-Inspired Robotic Manipulation Design

When tackling the robotic design of manipulation tools that are strongly characterized by capability in versatile manipulation, a methodological approach consists of investigating how manipulative actions are performed in order to understand fundamental design features in terms of architecture choice or control modes. In particular, manipulative actions are often classified into a comprehensive and hierarchical structure, namely a taxonomy of manipulative actions, which might suggest insights for implementation. In the following, we mention some works that follow this approach.

One of the basic manipulations is the grasping of an object. Cutkosky [7] developed a comprehensive and structured taxonomy of human grasps from video recordings of machinists performing manufacturing operations, later extended by Feix [8]. Bullock [9] built a taxonomy of grasps that integrates Cutkosky’s [7] and Feix’s [8] taxonomies of grasps and used it to analyze the frequency of occurrence of the grasps and their duration during manufacturing and housekeeping operations: the authors identified a group of 10 grasps that are the most frequent in real-world settings, and highlighted the dependence of the selected grasp type on the object to be manipulated [10] and on the task to be performed [11,12]. The role of the task in the choice of a grasp suggests that the study of a manipulative action in a specific application should not be isolated, but clearly framed within a manipulation sequence that includes the subsequent task. Several examples of fieldwork that employed this approach of identifying grasp or manipulation primitives from real manipulations to guide design are reported in the literature [13,14,15]. In Mizera et al. [15], in particular, a systematic analysis of manipulations for deep-sea archeology was conducted on a database of actions performed by archeologists in a simulated scenario. A taxonomy of manipulative actions was compiled to guide engineers in the development of appropriate architecture for a deep-sea robotic hand for archeological operation.

A comprehensive taxonomy of manipulative actions for the human hand was also proposed by Bullock [16], which discriminates actions according to contact with the object, prehension, and relative position and motion between the hand and the object. Contact is not surprisingly a criterion for analysis of the manipulation: in neurophysiology, the initiation of a contact event at the sensory level is deemed to separate different subgoals within a task, and to serve as a checkpoint for task progression monitoring by comparison with the predicted sensory event according to the internal model [17].

Contact discrimination was exploited recently in an automatic algorithm for semantic decomposition and recognition of manipulative actions, called semantic event chain (SEC) [18]. This algorithm refers to the ontology of human hand manipulative actions proposed in Wörgötter et al. [19], where actions are grouped according to goal similarity among “rearrange”, “destroy”, “take down”, “break”, “construct” and “hide” actions. For each of the instantiations of human hand actions (e.g., “cut”, “squash”, “draw”, “scoop”, “push”, “pick apart”, “put together”, etc.) the authors highlighted the shape the hand should have to be able to perform the action, and its requirements in terms of control. This approach offers a model to systematically translate needs into requirements for the system to be designed or implemented.

### 1.2. Rationales and Objectives of the Proposed Investigation

The reasons for choosing a specific manipulation procedure or tool for marine sampling with a remotely operated vehicle (ROV) has never been systematically described, and this knowledge is still limited to those who have personally taken part in ROV cruises. To the best of our knowledge, a book chapter by Clark et al. [20] on deep-sea biological sample collection is one of the few reports covering scientific sampling with ROVs. The lack of a systematic treatise on this topic is mostly due to the adoption of a trial-and-error approach for selecting the right procedure: this makes generalization hard until a reasonable amount of diverse evidence is collected, compared and organized according to the general principles that guided the choice.

For these reasons, we performed an investigation concerning biological sample collection procedures. Investigations can be performed with different methodologies. For example, in Mizera et al. [15] this analysis was tackled by asking researchers or divers to perform actions typical of marine archeological collection procedures in a simulated collection scenario in the laboratory. We thought that the adoption of this methodology in our case could cause us to neglect important aspects of both the marine environment and the organisms that influence collection procedure. Hence, we decided to rely on researchers’ descriptions of their direct experience and on video documentation. Notably, some interesting information reported in Clark’s book chapter related to ROV sampling [20] originated from personal communications to the author from ROV operators and scientists. Such reports support the validity of the methodology we selected, consisting of interviews with experts.

Collection of reports of sampling procedures involved researchers that took part in sample collection campaigns; those descriptions were integrated with the analysis of video reports from [21]. Once this database was built, a standardized way to analyze the performed manipulations had to be selected. To frame our methodology within a well-structured background, we considered procedures used in studies aimed at the analysis, segmentation and extraction of manipulation features from a number of examples [19].

Our aim was primarily to identify the specific needs of deep-sea scientific sampling that could lead to the development of efficient and versatile technologies. The added value of our approach does not reside in the claim of a new technology that could potentially solve manipulation related issues, but in a clear statement of the needs that a versatile solution should cover.

### 1.3. Structure of the Paper

The contribution of this paper resides in the identification of task-related requirements for the design of an innovative gripper and shared control algorithms for assisting the pilot during deep-sea sampling.

This process required two steps, the results of which are reported in the present series of two papers. The first step consisted of a thorough analysis of the commercially available gripper technologies currently used for underwater collection of scientific samples, and a systematic review of the research status of underwater grippers, which we performed in [22]. As a second step, in this paper, we investigated the manipulative actions needed in underwater manipulation for the collection of scientific samples. In addition, we discussed the manipulative needs that have already been satisfied by the available technologies, and the unresolved points, in order to define the engineering requirements for the design of an underwater gripper for scientific sampling in the deep sea. The results of this paper could benefit the wider marine robotics community, with additional application in the design of shared control algorithms assisting the pilot during deep-sea sampling.

Consequently, the paper is organized as follows. In Section 2, procedures adopted to interview the researchers and operators involved in deep-sea sampling are described. In Section 3, we discuss the needs in underwater manipulation that arose from the interviews. In Section 4, we discuss whether and how those needs are satisfied by the available technology, and provide a formal list of engineering requirements for an underwater gripper for biological sample collection. In Section 5, conclusions are drawn.

## 2. Materials and Methods

In Section 2.1, we describe the structure of the survey that investigated the needs of researchers in deep-sea sampling for scientific purposes, how the survey was distributed within the research community, and how data were collected. In Section 2.2, we describe the tools we used for data analysis and, in Section 2.3, how data were organized and analyzed.

### 2.1. Investigation of Manipulative Needs from the Direct Experience of Researchers

The survey had a two-step structure: Step 1 involved a brief questionnaire, Step 2 a more in-depth questionnaire or interview. The brief questionnaire was sent to marine researchers from the institutes and universities listed in Table 1. The brief questionnaire was also sent to the communication offices of the large marine communities listed in Table 1. The cover letter of the survey requested that the survey be forwarded to any researcher in the field.

Step 1 consisted of a brief questionnaire, in which the researcher had to indicate:

- Role assumed in ROV cruise operations;
- Detailed list of samples collected;
- His/her availability for a second, more in-depth questionnaire or interview.

Researchers that expressed availability for further collaboration were invited to contribute to Step 2 of the survey, either through a questionnaire supplied by email or via an online interview depending on their preference. The series of questions was identical in both the Step 2 interview and the questionnaire, and had the following structure (the complete list of questions is reported in Annex A, Supplementary Materials):

1. Description of the sample and its consistency and deformation properties;
2. Surrounding environment;
3. ROV sampling methods: detachment, collection and release type;
4. Suggestions on the “ideal” tools, and the features of samples that should be preserved for analysis.

Lines were pre-filled with the list of samples inserted by the researcher in Step 1 to guide more specific answers for each type of sample. Both the questionnaire and the interview followed the same structure, and therefore data from the interviews and the questionnaires were easily merged for analysis.

### 2.2. Definitions: Robotic Manipulative Actions and Semantic Event Chain

The rationale for the taxonomic analysis of actions is to classify actions in an organic and discrete way. This is fundamental for identifying similarities among actions and dependencies of those actions on specific factors (related to the situation or the aim), hence allowing their grouping. Once this framework is built, it can be exploited to create technologies that better suit the application, and planning and control frameworks that automate the series of actions can be developed.

To classify actions, a series of definitions concerning the level of complexity of the action, the type of action according to its purpose, and the role of the agents involved in the manipulation were stated.

Coherently with Aksoy et al. [23], we adopted the following levels in the taxonomy of manipulative actions we built, from the general to the particular:

- **Activity** or **Manipulation sequence** is intended as an articulated sequence of manipulations involving multiple objects for a specific task (e.g., collection of a fragment of coral);
- **Atomic manipulation** or **atomic action**, intended as the specific interaction of the manipulator with an object (e.g., break a coral fragment, insert a coral fragment into the biobox);
- **Key-event** is an event delimited by two specific time points, which are defined by the instant in which any pair of agents (e.g., objects, manipulators, tools, etc.) involved in the activity makes contact, or releases the contact (e.g., from the moment the manipulator makes contact with the organism, to the moment in which the organism is detached from the sediment, that is, it releases contact with the sediment);
- **Manipulation primitive** is the basic component of a manipulation, and consists of a semantic expression that is the nearest to the robot command language (e.g., move, exert force, etc.).

The concept of a key-event introduces a temporal segmentation to the activity, which depends on the spatial relationship between the objects observed in terms of contact. When observing contacts among all involved objects, the ongoing atomic action can be recognized by referring to a vocabulary of atomic actions proposed by Wörgötter et al. [19]. Wörgötter et al. identified 23 basic instantiations of atomic manipulations that can be performed with the human hand, and grouped them as follows according to their goal category:

- **Hand-only actions**, where only the hand (or a grasped tool) acts on an object;
- **Separation actions**, where the hand manipulates an object to remove, detach or separate it from another object;
- **Release determined actions**, where the hand combines an object with a second target object.

Our analysis of underwater manipulations differs from that in Wörgötter et al. [19] because of their hypothesis on the properties of the manipulation: they deal with objects that do not have separate movable parts and are not deformable. The morphological variety of marine species prevents us from stating analogous properties for our objects of interest. Nonetheless, we still considered it useful to map the actions we identified to the taxonomy of manipulative actions of human hand from [19] in order to discuss our results.

Actions can be represented by means of SEC matrices [19,24]. The columns of the matrix refer to subsequent key-events identified by time instants in which a contact between two agents is created or released; the rows of the matrix refer to a pair of agents involved in the manipulation (e.g., object–manipulator, manipulator–tool, tool–object, etc.). Each cell of the matrix represents the state of contact or non-contact of the pair of agents. Once a vocabulary of the actions is obtained and an action is recognized, the SEC matrices are also informative for identification of the specific role of the object in the manipulation [25]. The object roles were identified in Aein et al. [24], and we adopted in our analysis only those definitions reported in Table 2. One should note that we conceived the word “action” used in those definitions as related to one or a few consequent atomic manipulations (further information regarding the definitions we adopted is given in Section 3.2).

### 2.3. Data Analysis

Answers to the questionnaires and interviews were initially reorganized by grouping the targeted organisms by phylum, and then describing all the possible manipulation sequences that were used for a particular phylum, also reporting the eventual tools employed. Features related to the organism, environment, available equipment or intended post-collection analysis guided the choice of a particular procedure, and hence they were also reported.

As described in Section 2.2, in order to build a taxonomy of manipulative actions, we referred to the standardization of action primitives described in [18,19,23,24].

Each manipulation sequence reported by the researchers was manually segmented using the algorithm explained in Aksoy et al. [18]. Briefly, we considered the objects involved in the manipulation, and built the basic sequence of key-events. Then, we listed all the manipulation primitives needed to create or release a contact, resulting in a new key-event. We assigned the roles of the objects in the manipulation. We split the manipulation sequence into atomic manipulations whenever the manipulative role of an object changed or whenever the manipulator was released from any contact. We then illustrated how the atomic manipulation could be combined with others to build possible manipulation sequences, referring to those that were actually described by the researchers. We finally framed those actions within the classification of manipulation instantiations proposed by Wörgötter et al. [19], classified according to their goal (see Section 3.2).

The manipulation sequence we targeted to build our taxonomy of manipulative actions was the activity of “sampling a marine organism or one of its parts with a robotic manipulator (SMO)”. SMO considered:

- the detachment of the organism from the substrate or the detachment of a fragment from the whole organism;
- the collection of the sample;
- the placement into the storage system.

This analysis of manipulation sequences tackles the placement, but it does not deal with preparation or opening of the container, nor corrective actions in case placing is not straightforward. More specifically, our analysis of SMO does not include:

- the operations performed by the manipulator pilot to prepare, readjust, or open the selected storage system; generally, these actions are performed just before the sampling;
- the operation of fitting the sample to the storage system, which is often needed where the dimensions of the container are not suitable for the sample;
- measures to counteract eventual buoyancy or floating out of the sample after insertion in the storage system, or the closing mechanism of the container.

## 3. Results

### 3.1. Representativeness of the Data Collected: Role of the Researchers and Geographical Area

A total of 44 researchers responded to the Step 1 questionnaire:

- Nine had never directly participated in a campaign in which samples were collected with a ROV;
- Eight declared they were not available for further collaboration;
- Seven were sent the Step 2 questionnaire;
- Twenty were contacted to schedule the Step 2 interview.

A total of 17 researchers responded to one or the other of the forms of Step 2. Hence, at the end of the data collection, we had two complete answers to the Step 2 questionnaire and 15 complete answers to the Step 2 interview.

**Figure 1:**
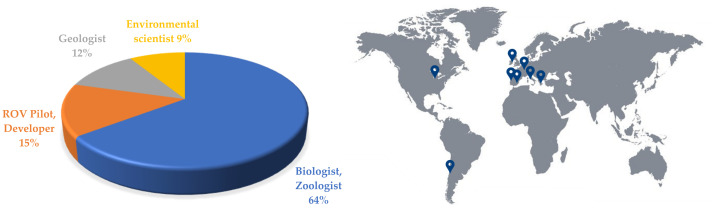
(**a**) Role of the researchers that participated in Step 2 questionnaires and interviews. (**b**) Geographic location of the institutions of affiliation of the researchers that participated in Step 2 questionnaires and interviews.

The roles of the researchers participating in Step 2 were biologists, zoologists, geologists, ROV pilots and developers (Figure 1a). The geographic spread of the institutions of affiliation of the interviewed researchers ensured variability of the samples of interest and independence of the answers in terms of the available equipment (vehicles and tools) for sampling or marine exploration (Figure 1b).

### 3.2. Taxonomy of Manipulative Actions for Marine Sampling Procedures

We parsed data collected on the sampling procedures described, and compared them with available online videos of ROV dives [21]. From this analysis, we identified a series of different atomic manipulations, reported in black in Table 3. We used the following notations to enumerate the actions. The first number is a progressive number for the basic action. The second character is:

- a number in case of a modified version of the basic action (e.g., basic action: *5. Grip*, *5.1 Grip and twist*);
- “A” if the action is performed using a tool; in this case, a third digit would distinguish among different tools (e.g., *3.A. Scoop with a tool*, *3.A.1 Scoop with scoop*, *3.A.2 Scoop with scoop-net*);
- “B” if the tool is the main (see Table 2) of the basic action.

In [22], we compared the atomic manipulations identified here from researcher evidence (Table 3, in black) with gripper technologies, to evaluate the possibility of action for each technology. Some of the grippers analyzed in [22] were tested for atomic manipulations that were not reported here by researchers; those atomic manipulations were subsequently added to our taxonomy and are reported in red in Table 3. This was the case for:

- *Suction on* and *off* performed without additional tools (atomic manipulations 7. and 9.): these atomic manipulations are just slightly modified versions of *Suction on* and *off with suction sampler* (atomic manipulations 7.A.1 and 9.A.1). Performing *Suction on* and *off* without an additional tool such as a suction sampler is possible if the gripper has a suction channel directly embedded into it.
- *Scissor cut*, *Lever* and *Pull (Hook)* (atomic manipulations 12., 13. and 14.): these manipulative actions extend the taxonomy we identified from the analysis of researcher reports. In researcher comments (see Section 4.2), there was mention of the importance of *Scissor cut* to avoid filamentous tissue tearing apart, and the use of the *Lever* action to detach protruding fragments of brittle rock, which is important for collecting biological samples that are anchored to that fragment.

**Figure 2:**
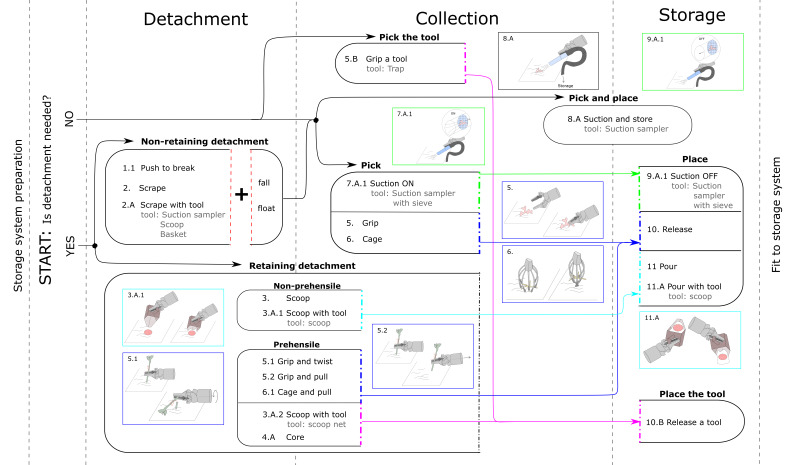
Taxonomy of atomic manipulations for marine sampling procedure with ROV. The arrows indicate how the atomic manipulations can be combined to build possible manipulation sequences.

Figure 2 illustrates how the atomic manipulations are combined in manipulation sequences. A complete solid line block represents a manipulation sequence that starts and ends with the gripper free from the main manipulated object. The arrows indicate possible combinations of atomic manipulations that build potentially feasible manipulation sequences. This figure does not include the atomic manipulations we added to our taxonomy from the results of [22], since those atomic actions were demonstrated while testing research prototype grippers, and those testing procedures did not necessarily consider the entire procedure of sampling a marine organism.

We preferred to limit the description of the manipulative sequences to procedures that are more consolidated within the common practice of sampling marine organisms. Extension of the manipulative sequence representation to new actions would need reports concerning complete sequences of sampling, in order to understand what are the applicable consecutive actions among those identified.

Annex B (Supplementary Materials) describes each of the manipulations identified with SEC matrices [19,24], following the methodology of the semantic event chain, and classifies the role each object assumes in the manipulation (see Table 2). It also lists the manipulation primitives needed to switch to the next key event. The execution of some of those primitives, such as *Exert force* or *Surface following*, would particularly benefit from a measure of force feedback. Other frequent primitives highlight the importance of the ability to turn the gripper or tool with a wrist-like degree of freedom (DoF), for example for the pouring action or for twisting to detach organisms or fragments.

Annex C (Supplementary Materials) reports the list of manipulation sequences suggested by researchers as the preferred pathway for the collection of a particular species or phylum, highlighting how the biological sample and features of the environment set the choice of manipulation sequence. The choice of a specific manipulation sequence according to these factors is discussed in Section 4.1.

The remaining outputs of the questionnaire are reported in Annex D (Supplementary Materials), which consists of the free-text comments from researchers concerning the grippers and tools they used during sampling procedures, and some additional desired features they would wish to integrate into an ideal tool. Those comments are discussed in Section 4.2.

### 3.3. Links to and Differences from the Ontology of Actions Identified for the Human Hand

The actions identified are easily framed within the ontology of atomic manipulations described by Wörgötter et al. [19] (see Section 2.2).

Before presenting how we linked our taxonomy to the ontology identified for the human hand, a disambiguation is needed concerning the way we classified the pick-and-place action, which slightly differs from [19]. Wörgötter et al. [19] state that an atomic manipulation starts and ends with the hand being free from any object (see rules no. 1 and 4 in [19]). Then, they describe a goal-based classification of the atomic manipulations, which distinguishes hand-only actions, separation actions and release-determined actions. They affirm that, on a closer look, the pick-and-place action is composed of a separation and a construction action. However, in order to preserve the “hand becomes free” condition, they preferred not to consider those sub-components as self-standing atomic manipulations, and to look at the pick-and place operation as a single release-determined atomic manipulation.

Within our taxonomy of manipulative actions, it might happen that the gripper contacts the object when detaching it, and the hand might not be free again until the storage phase is complete. Regardless, in the case of marine sampling, it seems too simplistic to consider this sequence a single release-determined atomic action as in Wörgötter et al. [19], for at least two reasons. Firstly, the sequence can be clearly divided into subcomponents (namely, the atomic manipulations) depending on its goal, which could be detachment, collection or storage. Secondly, as a matter of fact, most of the instantiation of detachment actions that we identified perfectly mirror some of the instantiation of hand-only actions proposed in [19]. Therefore, we decided to choose a stricter and goal-based perspective with which to look at the pick-and-place action, and consequently we separated it into subcomponents.

The framing of the actions of the underwater manipulation taxonomy into the ontology of actions identified for the human hand (Table 4) was fairly straightforward. Correspondence only failed to be found for the actions of suction, and hence these were added as new entries.

We also carried out, based on Wörgötter et al. [19], analysis of the features needed to perform each atomic action, in terms of manipulator shape, criticality of the approaching and acting trajectory, and relevance of positioning. The modification and additions we considered here are shown in bold in Table 4. The additions concern the “envelope” shape to enable the caging operation. The modification concerns the separation action pose that we labeled as “grasp-determined”, because constraints on the choice of the pose for collecting a sample are actually relevant and dictated by the properties of the organism itself and the subsequent task to be performed (generally, the storage part). This is a consequence of the way we conceived of the separation action, that is, as something that is directly followed by the release action without freeing the hand from the object.

## 4. Discussion

### 4.1. Preferred Grippers, Tools and Atomic Manipulations Considering Species Need

In this section, we analyze the manipulation strategy trends for each major phylum targeted, discussing the reports by researchers (Annex C): we try to highlight the role of factors which impact the choice of actions and tools, such as the type of sample (fragment, tissue, whole organism) and environment type.

#### 4.1.1. Cnidarians

Cnidarians [26] include a large variety of sessile (e.g., soft and hard-bodied solitary or colonial corals) and pelagic (e.g., jellyfishes) organisms with dimensions ranging from a few centimeters to a few meters. Moreover, in the case of branched coral (e.g., Octocorallia), one might decide to sample either a fragment or the whole organism, depending on the analysis to be performed. Nevertheless, we identified some common patterns for their collection at sea.

When the desired sample is a fragment of an organism with a brittle deformation characteristic (e.g., scleratinians), the choices are picking and breaking (by applying grip force, or by twisting the gripper), or scraping the branch tips with a tool that might eventually collect detached pieces before they fall; pushing branches to break them in order to collect fragments later is also a solution. For fragments of cnidarians that have a wiry skeleton and/or strong coenenchyme (e.g., some antipatharians such as *Leiopathes* sp., and some alcyonaceans), the applied force might fail to sever the skeleton, or might break the skeleton but not separate the tissues, and the overall gripping action might result in a squashed sample: therefore, researchers suggested implementing a cutting tool on the gripper, which could be activated to cut a branch or a fragment once the sample has been gripped.

When the whole colony (whole branching structure) is needed, the choice depends on a compromise between organism consistency and the consistency of the substrate. If the substrate is soft or loose, grasping the lowest visible part of the skeleton and pulling might work, since this will likely retrieve the whole organism, including the buried part. For brittle organisms, grasping the lowest visible part of the skeleton before pulling is important to avoid crumbling of the sample (e.g., some species of *Acanella*). Where brittle organisms (e.g., some species of *Acanella*, *Chrysogorgia*) or wiry elastic alcyonaceans are on friable substrate (such as dead framework coral or friable rock), pushing and breaking the substrate and then grasping the lowest visible part of the skeleton of the focal species is an effective strategy. For antipatharians that generally grow on harder substrates, which are difficult to break, grasping the lowest visible part of the skeleton and pulling might be the only choice to try to collect the whole organism, but strong pulling force is required.

Encrusting cnidarian species (e.g., zoantharians) might be scraped from their substrate, or a piece of the host structure could be collected if possible.

Some cnidarian taxa, such as sea anemones, sea pens, and tube-dwelling anemones (ceriantharians), might retreat into their tube or substrate, so they need to be approached with caution. In particular, the gripper should be fast-closing to allow the organism to be gripped before it retracts. Sea pens may have spines: in this case, the use of a suction sampler should be avoided, as it could lead to self-piercing and spoil the sample.

In our survey, jellyfish sampling was not addressed by the researchers. Deep-sea sampling of jellyfishes using ROV suction tools has been reported in the literature, mainly using suction samplers with gentle suction flow [27,28].

As pointed out in [22], research prototypes of grippers successfully demonstrated cnidarian collection. *Dendronephthya* and scleractinians were collected with the bellow-type actuator, and an alcyonacean whip coral with the boa-type [29,30]; both grippers implemented a scissor-like cutting tool. Three scyphozoans were collected with the Wyss Institute Ultragentle gripper [31], and a *Stellamedusa* collection directly from the water column was performed with RAD [32].

#### 4.1.2. Arthropods

The dimensions of marine arthropod [26] taxa such as copepods, amphipods or ostracods are measured on a millimetric or sub-millimetric scale, so their dimensions are quite small compared to the target object dimensions of ROV grippers. Researchers reported these organisms could hardly be seen before collection, and their sampling is possible via collection of host organisms or suctioning some substrate materials where they are likely to be found.

Other taxa of this phylum are instead much larger (at the centimeter or even meter scale, ranging from shrimps and lobsters to iconic species as the giant Japanese spider crab *Macrocheira*). Preferences among collection procedures depend mostly on size and lifestyle: pycnogonids of a few centimeters in size on soft substrates are well collected with sediment coring. Encrusting barnacles instead need to be detached from the substrate. Larger swimmers or crawlers such as crabs or shrimps are collected with traps, because the manipulator–gripper system is too slow to catch them in the water column (for example, in the case of tail flipping avoidance movements). The suction sampler might be used for organisms of sizes compatible with the sampler nozzle; for larger sizes, their rigid carapace could block the tube, making the sampler device unusable for the rest of the dive. Additionally, it was reported that when storage jars have a metallic filter at the end, the turning flux in the jars during suction could scratch the exoskeletons of arthropods.

#### 4.1.3. Mollusks and Echinoderms

Mollusks [26] mentioned during the interview were either shelled organisms (e.g., clams) of several centimeters or shell-less organisms, such as cephalopods (e.g., octopuses and squids) and worm-like aplacophorans. The shelled organisms, when buried or attached to the substrate, were scraped away, directly picked with suction, and finally stored. Alternatively, if these organisms lived in soft sediments, they were collected using push corers. Bivalves and aplacophorans were collected with traps. Aplacophorans have no shell and quite a soft consistency, hence, they were also reported to have been detached by scraping and then collected with a suction sampler or by scooping. A slow-moving octopus was reported to be picked with the claw, although this is generally not possible for fast moving organisms. Indeed, suction procedures, or the use of nets and traps, are mentioned in the literature as sampling methods for cephalopods [33,34]. Among research grippers presented in [22], shell collection was reported with the soft OBSS gripper [35], the Tshingua University cage gripper [36] was able to collect some scallops, and collection of *Oegospina* sp. and *Stigmatoteuthis* sp. squids was demonstrated with the RAD cage gripper [32].

Different collection strategies were reported for the echinoderms [26] according to class. Sea urchins (centimeter-scale in diameter) have characteristic spines that should be preserved during collection, and this is difficult to achieve by tuning the picking grasping force. Therefore, urchins are sometime scooped together with part of the substrate. Sea urchins have been successfully collected with the OBSS gripper [35] and the Tshingua University cage gripper [36]. Sea stars (generally several centimeters in diameter, up to one meter in diameter in the case of *Midgardia xandaros*) are picked or scooped as well, but due to their use of arm autotomy as a defense mechanism, the choice of grip location is fundamental. Sea stars were successfully collected with the Wyss 3DP gripper [37]. Sea cucumbers are up to a few tens of centimeters in length and have a soft to medium consistency and bodily turgidity. They can be taken with suction when small enough, or alternatively by scooping or picking. Sea cucumbers were collected with the OBSS gripper [35], Tshingua University cage gripper [36], Wyss 3DP gripper [37] and five-fingered bellow-type v2 actuator [30]. Finally, crinoids (approximately ten centimeters to one meter in length) were reported to be collected by grabbing and pulling, and then stored with suction sampler with a lowered suction flow (to avoid breaking them into pieces). Crinoids were successfully collected with the Wyss 3DP gripper [37].

#### 4.1.4. Porifera

Collection procedures for sponges [26] depend on the desired sample. For biological taxonomy analysis, the entire organism is needed, including root tufts or peduncles. In this case the technique consists of breaking and collecting the piece of substrate whenever possible, or coring when the sample is on a soft substrate. Other techniques consist of grabbing the sponge from the base and pulling, but this does not ensure sample integrity, especially when the substrate is hard. Moreover, applying the right amount of force to pull but not squeeze a sponge is a difficult operation. For encrusting sponges, if it is not possible to collect the rock itself, tissue is picked or scraped from the rock. Sponges have been collected with a Wyss 3DP gripper [37] and three- and two-fingered bellow-type v2 actuators [30].

#### 4.1.5. Other Phyla

Polychaetae worms [26] can be collected with a suction sampler, or by picking animals with the claw or coring. Cage grippers work successfully for tunicates [26] because these grippers can penetrate the substrate, such as the LIROPUS 2000 ROV gripper [22] (p. 6, Figure 1b). Scooping is also a good way to collect tunicates. Tunicates are very soft: during picking, as for sponges, tuning the force to pull but not squeeze the organism is difficult. Pyrosome collection was demonstrated with a three-fingered bellow-type v2 actuator [30], suggesting that some grippers can also be used to collect pelagic organisms characterized by slow movement. Other worm-like organisms such as Enteropneusta or Sipuncula [26] were collected through coring, while a custom rake system was reported for Bryozoa [26] collection. A bacterial mat (i.e., *Thiolava veneris*, that forms filamentous mats) was sampled by collecting the volcanic rocks on which this grew. Although not deep-sea, researchers also reported that algae [26] could be detached by pulling them, or by taking the rock to which they were attached.

### 4.2. Researchers’ Perspective: Pros and Cons of the Tools Used during Sampling Procedures

In this section, we present a summary and discuss the issues raised and the perspectives offered in the free-text comments of researchers concerning grippers and tools they used during sampling procedures.

#### 4.2.1. Tools Commonly Used during Sampling Procedures

Most of the comments on the grippers concerned the grasping force: not all devices provide force feedback to the pilot, and regulating the grasping force ends up being a difficult operation, highly reliant on the pilot’s experience. The issue of preventing unwanted release of the sample due to currents or during drag was often mentioned as well. Dimensions and closure range were two other important features: most of the animals, except for large benthic animals or rocks, would require a smaller gripper, or the possibility of having the full range closed (sometimes the samples slide through the fixture for the T-bar handle); on the other hand, full closure of the gripper could squash the sample. The closure speed of the gripper is equally important for samples that could escape or retract.

Comments on the suction sampler mentioned the issue of clogging: the choice of suction sampler for a sample is made looking at the compromise between suitable sample dimensions and consistency. In fact, big samples or very rigid ones are likely to block the tube. Once the sampler is clogged, it cannot be used for the remaining duration of the dive. Suction flow needs to be adjustable, because higher intensity is needed to detach the sample, but lower intensity flows prevent the loss of sample integrity for very fragile samples. Samplers need a good positioning system as well: positioning the tip close to the sample is crucial to obtain enough force for detachment, and a manipulator with good workspace and manipulability is important to use the sampler to approach organisms in the water column.

Problems related to storage systems can depend on the choices made during pre-dive. It is not easy to foresee the right container dimension or box partitioning, as this requires guessing which samples are likely to be found during the next dive. Moreover, the ROV might lack a built-in storage rack: in this case, the positioning of the basket container needs to be carefully chosen with respect to the manipulator’s position. In any case, accessibility of the container should always be checked in pre-dive. The insertion of the samples into a container is not an easy procedure either, but it is structured enough to be the best suited for automation, especially for complex actions such as pouring into a container. A frequent problem concerns the use of multiple partitions within the same box, because when the cover of a box is opened a second time for the insertion of a new sample, the previously inserted samples might float out because of buoyancy, ROV stabilization movements, or currents.

#### 4.2.2. Additional Desired Features on the Ideal Tool

The answers to the questions regarding desired features for an ideal gripping tool highlighted additional fundamental and optional features. First of all, a general-purpose tool was preferred to task-specific tools, because changing tools resting in the ROV rack generally takes time and tools can become tangled. While an envelope grasp was suggested for delicate animals (ideally, adding a fine sieve envelope to preserve microorganism populations), the need for a prehensile gripper remains for detachment purposes. Tweezer-like extensions to the fingers of common grippers were suggested for dealing with smaller samples. Similarly, the need for rigid parts for scraping, and concave parts for scooping, was underlined. Suction sampler functionality could be enhanced with the possibility of tuning suction flow, and a pruner-like cutting tool would be ideal for the highly frequent sampling of corals. For corers, a means of automation of the insertion or retrieval procedure that preserves substrate stratification is desirable, especially for geological analysis.

### 4.3. Technical Requirements for Grippers

In this section, we list the requirements for a gripper for biological marine sampling: we distinguish among environmental requirements, operational requirements and task-related requirements. Environmental requirements (Section 4.3.1) enable the technology to function underwater and withstand the typical issues of the marine environment. Operational requirements (Section 4.3.2) tackle the integration and compatibility of the technology with the instrumentation that supports its functioning, in this case the ROV system and its manipulator. Task-related requirements (Section 4.3.3) deal with constraints and desired features imposed by the activity to be performed, in this case “sampling a marine organism or one of its parts with a robotic manipulator (SMO)”.

#### 4.3.1. Environmental Requirements

Along with the study of the literature on underwater grippers performed in [22] and from the reports of the researchers regarding the sampling procedures analyzed in this paper, environmental requirements for a gripper for underwater manipulation were identified (Table 5).

Guaranteeing water resistance is normally tackled by exposing all mechanical components whose functioning is not hampered by the water, and enclosing only the water-sensitive ones (often motors and electronics) in water-resistant containers. Proper sealing techniques are available for different working depths and pressure. Water resistance is one of the main reasons why, in most of the designs, transmissions are employed to delocalize the actuators and control electronics from the distal part of the gripper. Issues in fabricating water-resistant electronic components affect the realization of functional tactile sensing for the marine environment (several functional reports are listed in Subad et al. [38]).

Tactile sensing functioning depends on pressure resistance: sensors should ideally work under a wide range of pressures while providing measurements that are independent from external pressure. In general, pressure-related issues are twofold: pressure adds design constraints to avoid the failure of the component, and it can affect water resistance by promoting water intrusion. Again, countermeasures are taken only for components sensitive to compression. Consistent with the expected working depth, countermeasures mainly involve:

- proper design and dimensioning of the component, including minimization of uncompensated volumes (e.g., being as tiny as possible when designing pressure resistant containers);
- pressurization of the components (e.g., filling components or actuators with incompressible oil; for hydraulically actuated systems, balancing external pressure; etc.).

Ideally, the specific gravity of the gripper should be near to that of water, so that its weight is mostly supported by upthrust (i.e., the material used for RAD has a specific gravity of 1.15). Moreover, internal volume variation among different configurations of the gripper should be avoided in cases of pressurized components (unless “adaptive” pressurization systems are available). Internal volume variation changes the upthrust effect, after which variation avoidance is needed to achieve accurate modeling and control against gripper buoyancy effects.

Recently implemented safety measures against mechanical overload or impacts consist of employing compliance, both intrinsic (as for soft technologies) and mechanical (as for backdrivable transmissions or decoupling actuation systems), and compliance in control systems, where the possibility of sensing is available.

#### 4.3.2. Operational Requirements

Along with the study of the literature we performed in [22] and from the reports of the researchers regarding sampling procedures analyzed in this paper, operational requirements for a gripper for underwater manipulation were identified (Table 6).

Considering that part of the weight of the gripper is also supported by upthrust, constraints on gripper mass related to arm payload requirements in general are not particularly stringent for hydraulically actuated manipulators, while they start becoming relevant for lightweight electronic manipulators. To reduce arm inertia, it is better to keep the mass as low as possible. This is one of the main reasons why under-actuation is selected for grippers: indeed, motors generally have a consistent weight.

Efficiency of energy use is important, especially where use on untethered systems is foreseen. To ensure low impact on the energy consumption of the supporting system (for discussion of power consumption for ROVs, see [39]), ideally the power consumption of the gripper should be an order of magnitude lower than the amount of consumption of the robotic arm that supports it (this ranges from hundreds of watts for lightweight arms to 10 kW for high-end hydraulic manipulators) [30,40].

#### 4.3.3. Task-Related Requirements

Based on analysis of the task (SMO) from researcher reports on sampling procedures that were analyzed in this paper, we extracted task-related fundamental and optional requirements (Table 7).

#### 4.3.4. Solutions That Sufficiently Satisfied Task-Requirements and Open Points

A first discussion point arises on the most suitable technology: a rigid gripper is a solution that generally satisfies RE.F T5. On the other hand, if the system is soft, it offers the possibility to absorb accidental impacts with the environment and to adapt its grasp to the organisms [38].

A prehensile grasp emerged as an important feature for detachment. Whenever collection of the sediment is not possible, and cutting part of the organism would prevent the intended analysis, the strategy is to try to pull the sample out from the sediment. In this case, the request for proper tuning of the grasping force for soft organisms or samples is dependent not only on the consistency of the organism but also on the amount of pulling force required to pull out the organism without squashing it. Force feedback is provided by few technologies, and currently most of the ability to control force depends on the pilot’s experience. Advances in sensor technology [38] that achieve pressure-independent reliable sensing solutions might drive attention toward addressing the implementation of controls for the automatic tuning of force. To this aim, a system that is fast and precise in the execution of commands is ideal. On one hand, hydraulic actuation has a higher power-to-weight ratio, but it is less suited for precise control than electrical actuation. On the other hand, the force requirements to collect soft specimens are lower than those required for intervention tasks. For these reasons, waterproofed electrical actuation could be a good choice for a gripper platform, in line with recent trends. This choice enables the implementation and testing of control algorithms for automatic force tuning, provided a suitable power supply and proper communication structures are available on the supporting ROV.

Combining multiple tools into a single instrument is advantageous from the perspective of saving time spent changing instruments, but might eventually require additional actuators to switch between the different configurations. A compromise between this integration and the number of controllable actuators should be envisioned, as well as a stable way to quickly and rigidly connect and disconnect tools that are not integrated into the gripper. The Victor 6000 claw [41], with its hollow components, is an example of the integration of a scoop into the system. Nonetheless, the grabber shape introduces some limitations in its picking ability. The IIT tool changing system [42] is illustrative of the concept of quick connection and disconnection.

Issues envisioned in the automation of the storage procedure concern both the gripper and manipulator systems. In teleoperation, imprecise absolute positioning of the manipulator might not be that relevant because the teleoperator might compensate for it, but it becomes significant when automated actions are implemented [40]; hence, precise positional control of the manipulator–gripper system is needed.

Finally, the payload requirements of the gripping system are mainly driven by the amount of force needed for performing the actions that are propaedeutic for sampling procedures. Typical payloads of commercial manipulator–gripper systems range from 10 kg to 40 kg for electrically actuated ones, and 10 kg to 500 kg for hydraulically actuated ones [40]. Gripper payload could be lowered compared to the common payloads in heavy industrial tasks, but this would limit the possibilities of sampling heavy rocks for geological analysis. Such samples are generally heavier and harder than biological ones. Commercial devices would likely be able to collect rocks, since the payload and torque requirements of rock collection are in line with the capabilities of industrial underwater manipulator–gripper systems. On the other hand, other aspects of geological analysis would be well served by improvements in tool positioning resulting in more precise gas collection, and the automation of corer storage would increase the likelihood of preserving stratification.

## 5. Conclusions

Manipulator–gripper systems for underwater operations were mainly conceived for the heavy duties of industrial and commercial applications, such as pipe inspection, cable management or sunken object recovery. While adaptation of the vision and sensing systems of industrial ROVs to scientific aims is more straightforward, the feasibility of tasks such as sample collection is limited, and requires non-standard equipment to be added. Moreover, equipment has to meet rigorous safety standards. Consequently, the longer-term perspective on augmentation of industrial ROVs with functional equipment for scientific purposes is dependent on a comprehensive presentation of the requirements in advance of the actual possibility of final deployment [43].

With this paper, we aimed to define the requirements for underwater biological sample collection in at least two respects. Firstly, a methodological analysis of the procedures for collection as explained by researchers in the field was performed to abstract the basic actions needed, regardless of the particular tool employed. A second approach considered further needs raised by the researchers, in order to improve the collection procedure, enable additional possibilities for sample analysis, or meet species needs.

The challenges in building a system that meets the requirements listed is related not only to the obstacles imposed by immersion in deep water, such as waterproofing or pressurization, but also to the fact the ideal device seems to be an integrated multi-purpose tool that is able to handle multiple situations that might be encountered in unpredictable and unstructured environments, demonstrating robustness and reliability. Complementary to improvements to tools for gripping and storage systems, the automation of structured but demanding tasks is relevant.

In our investigation, we had few reports on sampling procedures within the water column: this might have caused us to neglect specific actions for sampling in the water column in our taxonomy of manipulative actions. Further data collection in this area might extend this taxonomy.

The ultimate benefits of a system that enhances possibilities for scientific marine research are the reduction of the duration of diving campaigns, the reduction of pilot efforts and, not least, successful sampling. This drives toward a smarter employment of the resources available for exploration and direct analysis of the biodiversity of the deep ocean, which is still widely unexplored. Finally, enhancing manipulator dexterity and capabilities results in a greater chance of preserving an environment to which humans had limited access until a few decades ago. Moreover, those advances pave the way towards performing in situ analyses, or tests that would be influenced by bringing the sample to the surface.

## Figures and Tables

**Table 1:** Institutes, universities and marine communities contacted for Step 1 questionnaire.

**Institute/University**	**Acronym**	**Country**
Stazione Zoologica Anton Dohrn Napoli	SZN	Italy
Istituto di Scienze Marine del Consiglio Nazionale delle Ricerche	ISMAR-CNR	Italy
Istituto Superiore per la Protezione e la Ricerca Ambientale	ISPRA	Italy
Institute of Marine Sciences of Consejo Superior de Investigaciones Científicas	ICM-CSIC	Spain
University of Barcelona	UB	Spain
Universitat Politècnica de Catalunya	UPC	Spain
Institut Français de Recherche pour l’Exploitation de la Mer	IFREMER	France
Institut Méditerranéen d’Océanologie	MIO-OSUPYTHEAS	France
Sorbonne University		France
Helmholtz Centre for Ocean Research	GEOMAR	Germany
Hellenic Centre for Marine Research	HCMR	Greece
University of Victoria	UVIC	Canada
Ocean Networks Canada	ONC	Canada
**Marine community**	**Acronym**	**Country**
National Oceanic and Atmospheric Administration	NOAA	USA
International Network for scientific investigation of Deep-sea ecosystems	IN-DEEP	UK
Deep Ocean Stewardship Initiative	DOSI	UK

**Table 2:** Role of the objects in an atomic manipulation.

Role	Description
Manipulator	The agent that performs the action; during the action, it makes contact with at least one other agent, while it is free at the beginning and the end of action.
Main	The agent that is directly in contact with the *manipulator* (or *tool*); it is not touching the *manipulator* (or *tool*) at the beginning and the end of the action. It touches the *manipulator* (or *tool*) at least once.
Primary	The agent from which the *main* separates; it is initially in contact with the *main*, and there is a transition in which it breaks contact with the *main*.
Secondary	The agent to which the *main* joins; it is not initially in contact with the *main*, and there is a transition during which it makes contact with the *main*.
Main support	The agent upon which the *main* is located; it is touching the *main* object at all times.
Primary support	The agent upon which the *primary* is located; it is touching the *primary* at all times.
Secondary support	The agent upon which the *secondary* is located; it is touching the *secondary* at all times.
Tool	The agent used by the *manipulator* to enhance the quality of certain actions. It is grasped by the *manipulator* at the beginning of the action and released at the end.

**Table 3:** Taxonomy of manipulative actions for marine sampling with ROV. Each record in this table is an atomic manipulation. s.s. stands for suction sampler. In red, additional actions identified in [22].

Detachment	Collection	Storage
	8.A Suction and store	
1.1 Push to Break (+fall, +float)	5.B Grip a tool	
2. Scrape (+fall, +float)	7. Suction on	
2.A Scrape with tool (+fall, +float)	7.A.1 Suction on with s.s.	9. Suction off
12. Scissor cut	5. Grip	9.A.1 Suction off with s.s.
13. Lever	6. Cage	10. Release
3. Scoop		10.B Release a tool
3.A.1 Scoop with scoop		11. Pour
3.A.2 Scoop with scoop net		11.A Pour with tool
4.A Core		
5.1 Grip and twist		
5.2 Grip and pull		
6.1 Cage and pull		
14. Pull (Hook)		

**Table 4:** Framing taxonomy of underwater (UW) manipulative actions into the human hand ontology of action identified in [19]. Action 8.A is composed of a first part “Suction and …” classified as a hand-only action, and a second part “… and store” classified as a release-determined action. In **bold**, the modification and additions introduced in the analysis of the manipulation features with respect to [19].

Type	Hand Ontology Instantiation	UW Taxonomy Instantiation	Manipulator Shape	Trajectory		Pose
				Approach	Act	
Hand-Only actions	5. Turn = Bore	4.A Core	Point	Kinematic	Critical	Relevant
	7. Scratch	2. Scrape	Point			
		2.A Scrape with tool				
	8. Scissor-cut	12. Scissor cut	Edge			
	11. Push/Pull w/o grasp	1.1 Push to break 14. Pull (Hook)	Flat/Fist Hook			
	15. Lever	13. Lever	Flat			
	16. Scoop	3. Scoop	Hollow			
		3.A Scoop with tool				
		7. Suction on 7.A Suction on with tool	Suction flow	Kinematic /Dynamic	Critical	Relevant
		8.A Suction and store				
Separation actions	17. Pick apart	5. Grip	Grasp	Kinematic	Non- critical	**Grasp-** **determined**
		5.B Grip a tool				
		6. Cage	**Envelope**			
	19. Rip off 20. Break off	5.1 Grip and twist	Grasp		Critical	Grasp- determined
		5.2 Grip and pull				
	20. Break off	6.1 Cage and pull	**Envelope**			
Release determined actions	23. Put together	8.A Suction and store	Suction flow	Kinematic	Critical just before release	Relative pose to secondary object is relevant
		9. Suction off 9.A Suction off with tool				
		10. Release	Grasp			
		10.B Release the tool				
		11. Pour	Hollow			
		11.A Pour with tool				

**Table 5:** Environmental requirements.

Environmental Requirements:
Fundamental:
RE.F E1. The components selected for the gripper should be enclosed in water-resistant systems, or should be water-resistant by themselves, or should not lose their desired functioning in wet conditions. RE.F E2. The components selected for the gripper should be compression-insensitive or pressure-compensated. RE.F E3. The components selected for the gripper should be corrosion-resistant. RE.F E4. The gripper should be neutrally buoyant. RE.F E5. The gripper should counteract the displacement of the object that happens as the finger approaches an object in a fluid. RE.F E6. The gripper should be overall robust against mechanical overload and against (intentional or accidental) impact with the environment. RE.F E7. The working temperature of the gripper should include typical ocean temperatures of 0 °C to 3 °C.

**Table 6:** Operational requirements.

Operational Requirements:
Fundamental:
RE.F O1. The gripper should feature physical compatibility with the supporting manipulator. RE.F O2. The gripper actuation type should be compatible with that available on the supporting structure (i.e., hydraulic or electric) or the feasibility of the integration of new actuation types on board should be evaluated. RE.F O3. The gripper should comply with payload requirements of the arm and supporting structure. RE.F O4. The gripper should be lightweight, to avoid contributing to arm inertia. RE.F O5. The gripper control and sensing architecture should be compatible with the supporting structure, in terms of both communication protocols and physical interface (i.e., wired or wireless, CAN-bus), or the possibility to integrate additional architecture on board should be evaluated. RE.F O6. The gripper design should feature ease of maintenance and component substitution. RE.F O7. The gripper should be highly efficient in energy use.

**Table 7:** Task-related requirements.

Task-Related Requirements:
Fundamental:
RE.F T1. The gripper should be able to collect: organisms whose main dimensions range between a few centimeters and 15 cm; organisms under the centimeter scale down to microscale, possibly with some of the surrounding environment. RE.F T2. The gripper payload should account for the force needed to push an object into or pull it out of the marine sediment. RE.F T3. The gripper should be able to collect organisms at depths ranging from 50 m up to 4000 m. RE.F T4. The gripper should perform the following actions while minimizing changes of tool or gripper: Push to break, Scrape, Scoop, Core, Grip, Cage, Suction, Release, Pour, Scissor Cut, Hook, Lever. If tool changes are required to perform those actions, a modular and fast changing solution should be envisioned that does not limit the workspace of the manipulator. RE.F T4.1. The gripper should feature a hollow component to enable scooping. RE.F T4.2. The gripper should feature an enveloping component for caging: it could be either multiple fingers or a spherical component that can be closed when needed. RE.F T4.3. The gripper should feature a scissor cut tool mounted in series with respect to the claw, whose actuation is separated from the one of the claws. RE.F T4.4. The gripper should feature components needed for arm-controlled exertion of force, to execute actions as push to break or scrape. RE.F T4.5. The gripper should feature the possibility of turning the wrist (if not redundant with respect to that of the arm) for actions such as grip and twist or pour. RE.F T5. The gripper should be robust enough to penetrate soft or friable environments, to push against hard rocks, or to scrape along their surface. RE.F T6. The gripping system should have a claw architecture capable of performing prehensile grasps. RE.F T6.1. Prehensile claws should have high resolution in control of both position and force. RE.F T6.2. Prehensile claws should feedback force and contact information or automatically tune force. RE.F T6.3. Prehensile claws should feature fast and reactive closure. RE.F T6.4. Fingers and fingertip shapes should be tailored to work with objects with dimensions ranging from a few mm to 15 cm. RE.F T6.5. The internal side of fingers should have a surface that avoids object slippage. RE.F T6.6. The internal side of fingers should have a compliant surface. RE.F T7. The system should have a suction sampler. RE.F T7.1. The suction sampler should have an unclogging mechanism. RE.F T7.2. Suction flow should be tunable. RE.F T7.3. The suction sampler should have a removable sieve mechanism that prevents organisms from going through the tube when not desired. RE.F T8. The gripping system should have a rigid tool holding system for tools that are difficult to integrate (as the corer might be). RE.F T8.1. The gripping system should have a fast tool-changing system. RE.F T9. The design should minimize visual occlusions to the external camera. RE.F T10. Automation of sample storage procedures should be envisioned, with the possibility of triggering automatic algorithms for: RE.F T10.1. Insertion of samples into bioboxes. RE.F T10.2. Pouring from scoop or hollow components into bioboxes. RE.F T10.3. Recovery of the corer, while avoiding corer skew to preserve substrate stratification.
Optional:
RE.O T1. The gripper should be able to collect and manipulate organisms bigger than 15 cm. RE.O T2. The gripper should be able to collect and manipulate heavy, big, and irregularly shaped rocks. RE.O T3. The gripper should be able to collect organisms at depths ranging to 6000 m. RE.O T4. Tool-changing systems should not subtract space from the ROV rack. RE.O T5. The gripper should feature the possibility of enclosing the organism in a sieved envelope that retains particles bigger than 500 μm, so as not to lose the microorganisms living in the sample. RE.O T6. The suction sampler component with characteristics reported in RE.F T7 should be directly integrated into the gripping system.

## Data Availability

The data presented in this study are available in this Article and Supplementary Materials.

## Supplementary Materials

The following are available online at https://www.mdpi.com/article/10.3390/s22041471/s1: Annex A: Survey Structure [20,44,45,46,47]; Annex B: SEC analysis of the atomic manipulations; Annex C: Manipulation sequences and employed tools per species; Annex D: Comments on existing and ideal tools for sampling.
