# Supplementary material for: Marine Robotics for Deep-Sea Specimen Collection: A Taxonomy of Underwater Manipulative Actions
Source: Sensors (Basel). 2022 Feb 14;22(4):1471. doi: 10.3390/s22041471 (PMC8878465; doi:10.3390/s22041471)
Supplement: Supplementary file 1 [file sensors-22-01471-s001.zip › sensors-1539539-supplementary/Annex_A_Step2Questionnaire_v3.pdf]

Interview

Dear Researcher, thank you for having decided to help us in this project aimed at developing a gripper for ROVs for samples collection and processing.

The questions are targeted to the species/samples you mentioned in the survey as the ones you collected during a ROV campaign.

The species/samples you listed are (you can add other species you collected if you wish):

Sample A from Specie 1
Sample B from Specie 1
Specie 2

INSTRUCTION: Please, put at least a cross for each specie in yellow areas, or write free text in green areas. Please consider that the answers should refer to the samples you collected. Please answers to all the questions within this questionnaire. In case of doubt, or if you feel the labels are too strict for your case, please feel free to answer with free text.

If you find the questions are difficult to be answered because organisms within the same taxonomical name behaves differently, please use more specific taxonomy to refer to the samples you collected (eventually, you can add some rows).

In case you need more information, please contact me at angela.mazzeo@szn.it and I will be glad to help you. We can eventually switch to an interview, in which we can discuss together those questions.

Thanks for the help you are going to provide.

Size range of the organisms

**Size**	Micro/meio-fauna <0.5 mm	Macrofauna 0.5 mm to 50 mm	Megafauna >50 mm	If applicable, write approximatively the dimensions in mm (i.e. length x width x height)
Sample A from Specie 1	☐	☐	☐	
Sample B from Specie 1	☐	☐	☐	
Specie 2	☐	☐	☐	
	☐	☐	☐	
	☐	☐	☐	
	☐	☐	☐	

Notes: If you want, please add here details about the shape of the organism you collected.

Which depth did you find those samples?

**Depth**	m
Sample A from Specie 1	
Sample B from Specie 1	
Specie 2	

If you remember, please specify the model of ROV or robotic arm used

Notes: If you want, please add here details about the depth where you found the organism you collected.

Did you collect the whole organism directly, or just part of it, or something the organism was attached to?

Example for *Part of it*: branch or fragment of a coral instead of the whole coral tree.

We collected…	WHOLE ORGANISM	PART OF IT<br>Please, describe which part of the organism was collected and the dimension of this part in mm<br>(i.e. length x width x height)	THE ROCK/SEDIMENT/ANYTHING ELSE THAT THE ORGANISM WAS ATTACHED TO<br>Please, describe what was collected and the dimension of this part in mm (i.e. length x width x height)
Sample A from Specie 1	☐	☐	☐
Sample B from Specie 1	☐	☐	☐
Specie 2	☐	☐	☐
	☐	☐	☐
	☐	☐	☐
	☐	☐	☐

Notes: If you want, please add here details about the shape of the part you to collected.

Consistency: Is the organism or sample soft or rigid?

Please, try to describe the consistency of the organism.

**Consistency of the organism**	Very Soft	Soft	Medium	Rigid	Other (specify)
Sample A from Specie 1	☐	☐	☐	☐	☐
Sample B from Specie 1	☐	☐	☐	☐	☐
Specie 2	☐	☐	☐	☐	☐
	☐	☐	☐	☐	☐
	☐	☐	☐	☐	☐
	☐	☐	☐	☐	☐

In case you did not collected the sample directly, please describe also the consistency of the piece of rock/sediment/anything else the organism was attached to

Notes: If you want, please add details on the consistency of the sample you collected.

Consistency and deformation: What happens if you try to deform the organism or the sample?

**When deformed, warped, compressed or bent**	RIGID: It cannot be deformed	BRITTLE: It easily breaks into fragments	NON-REVERSIBLE: It does not go back to its original shape	REVERSIBLE: It goes back to original shape	OTHER: Please describe
Sample A from Specie 1	☐	☐	☐	☐	☐
Sample B from Specie 1	☐	☐	☐	☐	☐
Specie 2	☐	☐	☐	☐	☐
	☐	☐	☐	☐	☐
	☐	☐	☐	☐	☐
	☐	☐	☐	☐	☐

In case you did not collected the sample directly, please describe also the deformation behavior of the piece of rock/sediment/anything else the organism was attached to

Notes: If you want, please add details on what happens to the organisms when it is deformed.

Lifestyle of the organism

	Swimmer (floating)	Walker	Crawler	Sessile	Other, please describe
Sample A from Specie 1	☐	☐	☐	☐	
Sample B from Specie 1	☐	☐	☐	☐	
Specie 2	☐	☐	☐	☐	
	☐	☐	☐	☐	
	☐	☐	☐	☐	
	☐	☐	☐	☐	

Notes: If you want, please add details on the lifestyle of the organism you collected.

Habitat of the organism

**Habitat**	Water column	Seabed epibenthic	Seabed endobenthic	Other
Sample A from Specie 1	☐	☐	☐	
Sample B from Specie 1	☐	☐	☐	
Specie 2	☐	☐	☐	
	☐	☐	☐	
	☐	☐	☐	
	☐	☐	☐	

Notes: If you want, please add details on the habitat of the organism you collected.

Substrate

Substrate (if applicable)	Coastal	Ice	Benthic rock	Benthic sand	Benthic mud	Not applicable	Other (please specify)
Sample A from Specie 1	☐	☐	☐	☐	☐	☐	
Sample B from Specie 1	☐	☐	☐	☐	☐	☐	
Specie 2	☐	☐	☐	☐	☐	☐	
	☐	☐	☐	☐	☐	☐	
	☐	☐	☐	☐	☐	☐	
	☐	☐	☐	☐	☐	☐	

Notes: If you want, please add details about the substrate where the organism that you collected lived.

Ecosystem

Ecosystem	Hydrothermal vent	Seamount	Canyon	Wall/steep slope	Shelf/ slope flat ground	Cold seep	Mud volcano	Asphalt field	Nodule field	Other (please specify)
Sample A from Specie 1	☐	☐	☐	☐	☐	☐	☐	☐	☐	
Sample B from Specie 1	☐	☐	☐	☐	☐	☐	☐	☐	☐	
Specie 2	☐	☐	☐	☐	☐	☐	☐	☐	☐	
	☐	☐	☐	☐	☐	☐	☐	☐	☐	
	☐	☐	☐	☐	☐	☐	☐	☐	☐	
	☐	☐	☐	☐	☐	☐	☐	☐	☐	
	☐	☐	☐	☐	☐	☐	☐	☐	☐	

Notes or comments: If you want, please add details about the ecosystem where the organism that you collected lived in.

Reference Images for collection tools and storage containers

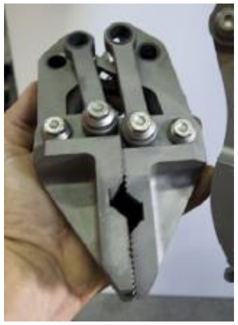

Fig. 1 Parallel jaw gripper

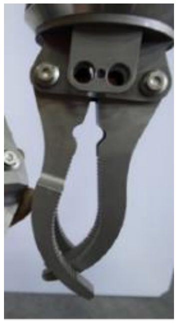

Fig. 2 Intermeshed fingers gripper

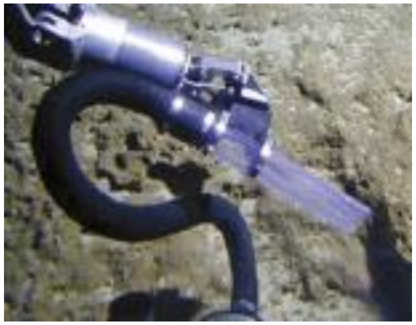

Fig. 3 Suction sampler

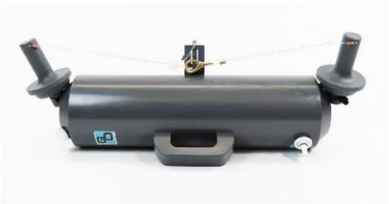

Fig. 4 Collection bottle (i.e. Niskin)

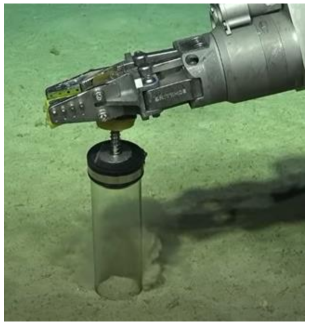

Fig. 5 Push or tube corer

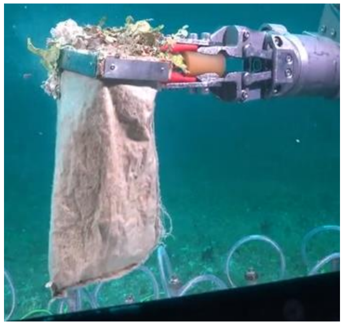

Fig. 6 Scoop net

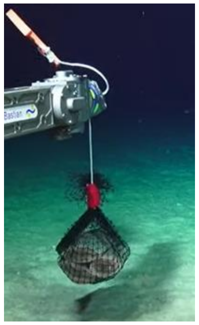

Fig. 7 Traps or buckets (previously left in the sea to capture organisms, or to be populated by them)

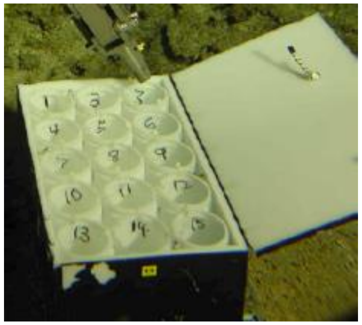

Fig. 8 Biobox

References:

Fig. 1, 2, 3, 8: © Wiley, reproduced with permission from:
Clark et al., 2016, Biological sampling in the deep sea, see [20]
Fig. 4: © General Oceanics, reproduced with permission from:
https://www.generaloceanics.com/rov-niskin-water-sampler-5l.html , see [44]
Fig. 5, 6, 7 © Schmidt Ocean Institute, reproduced with permission from:
https://www.youtube.com/watch?v=L_Ee7JV5oSI&t=704s , see [45]
https://youtu.be/ROp8UrUxATc?t=3783 , see [46]
https://youtu.be/zRDXpc_4gE8?t=2413 , see [47]

Collection through ROV: which collection tool was used?

**Collection tool**	Parallel Gripper	Intermeshed fingers gripper	Collecting bottle	Suction sampler	Scoop net	Tube or push corer	Traps or buckets (previously left in the sea to capture organisms/to be populated by organisms)	Other
Sample A from Specie 1	☐	☐	☐	☐	☐	☐	☐	
Sample B from Specie 1	☐	☐	☐	☐	☐	☐	☐	
Specie 2	☐	☐	☐	☐	☐	☐	☐	
	☐	☐	☐	☐	☐	☐	☐	
	☐	☐	☐	☐	☐	☐	☐	
	☐	☐	☐	☐	☐	☐	☐	

Notes or comments: Please detail here the sampling procedure (i.e. Collection of a trap, bait or bucket that was previously left in the sea to be populated by organisms).

If a gripper was used, its metallic fingers were used as they are, or covered with something softer (i.e. foam, silicone pad)?

Collection through ROV: did you need to detach the organism?

Please, if the sample was attached to a substrate, describe here the procedure and the tools used to detach it.

	Procedure to detach the sample
Sample A from Specie 1	
Sample B from Specie 1	
Specie 2	

Notes or comments:

Collection through ROV: Which storage container was used?

	Biobox	Collecting bottle	Carousel Jar	Scoop net	Tube or push corer	Trap	Other
Sample A from Specie 1	☐	☐	☐	☐	☐	☐	
Sample B from Specie 1	☐	☐	☐	☐	☐	☐	
Specie 2	☐	☐	☐	☐	☐	☐	
	☐	☐	☐	☐	☐	☐	
	☐	☐	☐	☐	☐	☐	
	☐	☐	☐	☐	☐	☐	

Notes or comments: Please describe here any particular feature of the containers you used

Collection through ROV: Was there any difficulty in the insertion of the sample into the storage container of the ROV?

Please, if it was difficult someway to insert the sample in the storage container in the drawer of the ROV, explain here why.

Sample A from Specie 1	
Sample B from Specie 1	
Specie 2	

Notes or comments:

How to improve the collection procedure: Why the gripper would not be completely suitable to collect the sample? (PLEASE, ANSWER TO THIS QUESTION EVEN IF THE GRIPPER WAS NOT ACTUALLY USED)

Suggest some reasons why - in your opinion - the gripper is not completely suitable to collect the sample.

Select or suggest at least one that applies for each specie (at least one cross per column), eventually adding other reasons not mentioned.

**Gripper is not suitable for…**	Sample A from Specie 1	Sample B from Specie 1	Specie 2			
… because the sample is too small to be taken with the gripper.	☐	☐	☐	☐	☐	☐
… because the sample is too big to be taken with the gripper.	☐	☐	☐	☐	☐	☐
… because the environment around the sample is too cluttered.	☐	☐	☐	☐	☐	☐
… because the sample escapes.	☐	☐	☐	☐	☐	☐
… because the sample can't be reached with the gripper, because of … (please complete)	☐	☐	☐	☐	☐	☐
… because it is difficult to tune the grasping force not to stress the delicate animal.	☐	☐	☐	☐	☐	☐
… because it is difficult to drag the animal into the box with the gripper without stressing it.	☐	☐	☐	☐	☐	☐
… because it is difficult to prevent unwanted release of the animal during drag to the storage drawer.	☐	☐	☐	☐	☐	☐
… because it is difficult to release the animal, because it sticks to the claw.	☐	☐	☐	☐	☐	☐
…	☐	☐	☐	☐	☐	☐
…	☐	☐	☐	☐	☐	☐
…	☐	☐	☐	☐	☐	☐
…	☐	☐	☐	☐	☐	☐

Notes or comments:

How to improve the collection procedure: Why the suction sampler would not be completely suitable to collect the sample? (PLEASE, ANSWER TO THIS QUESTION EVEN IF THE SUCTION SAMPLER WAS NOT ACTUALLY USED)

Suggest some reasons why - in your opinion - the suction sampler is not completely suitable to collect the sample.

Select or suggest at least one that applies for each specie (at least one cross per column), eventually adding other reasons not mentioned.

**Suction sampler is not suitable for…**	Sample A from Specie 1	Sample B from Specie 1	Specie 2			
… because the sample is too small to be taken with the suction sampler.	☐	☐	☐	☐	☐	☐
… because the sample is too big to be taken with the suction sampler.	☐	☐	☐	☐	☐	☐
… because the environment around the sample is too cluttered.	☐	☐	☐	☐	☐	☐
… because the sample escapes.	☐	☐	☐	☐	☐	☐
… because the sample can't be reached with the suction sampler, because of … (please complete)	☐	☐	☐	☐	☐	☐
… because the suction force would stress the animal.	☐	☐	☐	☐	☐	☐
… because it is difficult to bring it to the jar, because it sticks to the suction sampler tube.	☐	☐	☐	☐	☐	☐
…	☐	☐	☐	☐	☐	☐
…	☐	☐	☐	☐	☐	☐
…	☐	☐	☐	☐	☐	☐
…	☐	☐	☐	☐	☐	☐

Notes or comments:

How to improve the collection procedure: The ideal tool

How should an ideal tool be, to simplify the collection of each of those species? Please suggest any ideal feature, that would allow for sampling the organism/sample in the best way you can imagine.

i.e. To collect *this organism*, an ideal gripper should have a scissor to cut *this part* to avoid damages to *this other part*, and then collect *this fragment* extremely gently because it is very brittle.

**To collect…**	**…the ideal gripper should…**<br>Suggestions: should be… / should do…. (what?) / should sense…. (what?)/ should stop… (when?)
Sample A from Specie 1	
Sample B from Specie 1	
Specie 2	

Notes or comments:

In some cases, manipulative actions might alter the result of your intended analysis, or prevent them (some examples below in grey). Was there something you asked the pilot to pay particular attention to, in order not to alter your intended analyses? Which part of the organism you wanted not to be destroyed during manipulation? Which one were accidentally destroyed instead?

i.e. 1. Use of suction on a sample cleaned it from the microorganisms you were interested in; 2. You want to study the root part of a sessile specie, but during the detachment that part remained trapped into the substrate.

Sample A from Specie 1	
Sample B from Specie 1	
Specie 2	

Notes or comments:
