# Supplementary material for: Marine Robotics for Deep-Sea Specimen Collection: A Taxonomy of Underwater Manipulative Actions
Source: Sensors (Basel). 2022 Feb 14;22(4):1471. doi: 10.3390/s22041471 (PMC8878465; doi:10.3390/s22041471)
Supplement: Supplementary file 1 [file sensors-22-01471-s001.zip › sensors-1539539-supplementary/Annex_B_SEC_v07.pdf]

	Manipulative Actions	Manipulator	Tool	Objects role: Main	Primary	Main Support	Secondary	Semantic Event chain analysis and motion primitives: Contact between	Motion primitives
1.1	**Push to break**	claw<br>claw<br>claw	-<br>-<br>-	organism<br>fragment<br>fragment	substrate<br>organism<br>organism	water<br>water<br>water	substrate<br>substrate<br>organism	R Manipulator Main<br>R Main Primary<br>R Main Secondary<br>R Main Main Support	Manip -> Moves(Main)<br>Manip -> Preshape<br>Manip -> ExertForce(Main)<br>Manip -> Moves(home)<br>Main -> Float(Main support)
2	**Scrape**	claw<br>claw<br>claw	-<br>-<br>-	organism<br>fragment<br>fragment	substrate<br>organism<br>organism	water<br>water<br>water	substrate<br>substrate<br>organism	R Manip Main<br>R Main Pri<br>R Main Sec<br>R Main M_support	Manip -> Moves(Main)<br>Manip -> Preshape<br>Manip -> ExertForce(Main)<br>Manip -> SurfaceFollowing(Primary)<br>Manip -> Moves(home)<br>Main -> Float(Main support)
2.A	**Scrape with tool**	claw<br>claw<br>claw<br>claw<br>claw	suction sampler<br>suction sampler<br>suction sampler<br>scoop<br>basket	organism<br>fragment<br>fragment<br>organism<br>fragment	substrate<br>organism<br>organism<br>substrate<br>organism	water<br>water<br>water<br>water<br>water	substrate<br>substrate<br>organism<br>substrate<br>basket	R Manip Tool<br>R Tool Main<br>R Main Pri<br>R Main Sec<br>R Main M_support	Manip -> Moves(Tool)<br>Manip -> Preshape<br>Manip -> Grasp(Tool)<br>Manip -> Moves(Main)<br>Manip -> ExertForce(Main)<br>Manip -> SurfaceFollowing(Primary)<br>Manip -> Moves(home)<br>Main -> Float(Main support)
3	**Scoop**	Victor claw	-	organism and substrate	substrate	water		R Manip Main<br>R Main Pri<br>R Main Sec<br>R Main M_support	Manip -> Moves(Main)<br>Manip -> Preshape<br>Manip -> Exert force(Main, Primary)<br>Manip -> Turn<br>Manip -> Pull(home)
3.A.1	**Scoop with tool**	claw	scoop	organism, substrate	substrate	water		R Manip Tool<br>R Tool Main<br>R Main Pri<br>R Main Sec<br>R Main M_support	Manip -> Moves(Tool)<br>Manip -> Preshape<br>Manip -> Grasp(Tool)<br>Manip -> Moves(Main)<br>Manip -> Preshape<br>Manip -> Exert force (Main, Primary)<br>Manip -> Turn<br>Manip -> Pull(home)
3.A.1	**Scoop with tool**	claw	scoop net	organism, substrate	substrate	water	scoop net	R Manip Tool<br>R Tool Main<br>R Main Pri<br>R Main Sec<br>R Main M_support	Manip -> Moves(Tool)<br>Manip -> Preshape<br>Manip -> Grasp(Tool)<br>Manip -> Moves(Main)<br>Manip -> Preshape<br>Manip -> Exert force (Main, Primary)<br>Manip -> Turn<br>Manip -> Pull(home)
4.A	**Core with corer**	claw	corer	organism, substrate	substrate	water	corer	R Manip Tool<br>R Tool Main<br>R Main Pri<br>R Main Sec<br>R Main M_support	Manip -> Moves(Tool)<br>Manip -> Preshape<br>Manip -> Grasp(Tool)<br>Manip -> Moves(Main)<br>Manip -> Preshape<br>Manip -> Exert force (Main, Primary)<br>Manip -> Pull(home)
5	**Grip**	claw<br>claw	-<br>-	organism<br>fragment	substrate, water, organism<br>substrate, water, organism	water<br>water		R Manip Main<br>R Main Pri<br>R Main Sec<br>R Main M_support	Manip -> Moves(Main)<br>Manip -> Preshape<br>Manip -> Grasp (Main)<br>Manip -> Moves(home)
5.1	**Grip and twist**	claw	-	organism	substrate	water		R Manip Main<br>R Main Pri<br>R Main Sec<br>R Main M_support	Manip -> Moves(Main)<br>Manip -> Preshape<br>Manip -> Grasp (Main)<br>Manip -> Twist
5.2	**Grip and pull**	claw	-	organism	substrate	water		R Manip Main<br>R Main Pri<br>R Main Sec<br>R Main M_support	Manip -> Moves(Main)<br>Manip -> Preshape<br>Manip -> Grasp (Main)<br>Manip -> Pull(home)
5.B	**Grip tool**	claw	-	trap	substrate	water		R Manip Main<br>R Main Pri<br>R Main Sec<br>R Main M_support	Manip -> Moves(Main)<br>Manip -> Preshape<br>Manip -> Grasp (Main)<br>Manip -> Moves(home)
6	**Cage**	Liropus claw<br>Victor claw	-<br>-	organism, substrate<br>organism, substrate	substrate<br>substrate	water<br>water		R Manip Main<br>R Main Pri<br>R Main Sec<br>R Main M_support	Manip -> Moves(Main)<br>Manip -> Preshape<br>Manip -> Envelop (Main)<br>Manip -> Pull(home)

Notes

.+fall

Main -> Moves(Sec)

.+float

6.1	**Cage and pull**	Liropus claw<br>Victor claw	-<br>-	organism, substrate<br>organism, substrate	substrate<br>substrate	water<br>water		R Manip Main<br>R Main Pri<br>R Main Sec<br>R Main M_support<br>Manip -> Moves(Main)<br>Manip -> Preshape<br>Manip -> Envelop (Main)<br>Manip -> Pull(home)	
7.A	**Suction on with SS**	claw<br>claw	suction sampler with sieve<br>suction sampler with sieve	organism<br>fragment	substrate, water, organism<br>substrate, water, organism	water<br>water		R Manip Tool<br>R Tool Main<br>R Main Pri<br>R Main Sec<br>R Main M_support<br>Manip -> Moves(Tool)<br>Manip -> Preshape<br>Manip -> Grasp(Tool)<br>Manip -> Moves(Main)<br>Manip -> Turn on suction<br>Main ->Moves(Tool)	The double dot dashed region is needed only if the previous action was not made with SS
8.A	**Suction and store**	claw<br>claw	suction sampler<br>suction sampler	organism<br>fragment	substrate, water, organism<br>substrate, water, organism	water<br>water	jar<br>jar	R Manip Tool<br>R Tool Main<br>R Main Pri<br>R Main Sec<br>R Main M_support<br>Manip -> Moves(Tool)<br>Manip -> Preshape<br>Manip -> Grasp(Tool)<br>Manip -> Moves(Main)<br>Manip -> Turn on suction<br>Main->Moves(jar)	
9.A	**Suction off with SS**						biobox<br>drawer	R Manip Tool<br>R Tool Main<br>R Main Pri<br>R Main Sec<br>R Main M_support<br>Manip -> Moves(Secondary)<br>Manip -> Turn off suction<br>Main -> Float(Main support)	
10	**Release**						biobox<br>drawer	R Manip Main<br>R Main Pri<br>R Main Sec<br>R Main M_support<br>Manip -> Moves(Sec)<br>Manip -> Release<br>Manip -> Shaking<br>Main -> Float(Main support)	The double dot dashed region is needed only if the previous action was not made with SS
10.B	**Release tool**	claw<br>claw	-<br>-	scoop net<br>corer	claw<br>claw	water<br>water	drawer<br>drawer	R Manip Main<br>R Main Pri<br>R Main Sec<br>R Main M_support<br>Manip -> Moves(Secondary)<br>Manip -> Insert(Secondary)<br>Manip -> Release<br>Main -> Fall(Secondary)	The double dot dashed region is needed only if the previous action was not made with SS
11	**Pour**						biobox<br>drawer	R Manip Main<br>R Main Pri<br>R Main Sec<br>R Main M_support<br>Manip -> Moves(Seconday)<br>Manip -> Turn<br>Main -> Fall(Secondary)	
11.A	**Pour with tool**						biobox<br>drawer	R Manip Tool<br>R Tool Main<br>R Main Pri<br>R Main Sec<br>R Main M_support<br>Manip -> Moves(Sec)<br>Manip -> Turn<br>Main -> Fall(Secondary)	
10	**Scissor Cut**								
11	**Hook**								
12	**Lever**								
