# Supplementary material for: Marine Robotics for Deep-Sea Specimen Collection: A Taxonomy of Underwater Manipulative Actions
Source: Sensors (Basel). 2022 Feb 14;22(4):1471. doi: 10.3390/s22041471 (PMC8878465; doi:10.3390/s22041471)
Supplement: Supplementary file 1 [file sensors-22-01471-s001.zip › sensors-1539539-supplementary/Annex_C_ManipulativeSequencesPerSpecie.pdf]

Cnidarians

i.e.	Condition	Detach		Pick and place		Store	
Scleractinia<br>Antipatharia	SHAPE: Arborescent<br>CONS&DEF: Rigid and brittle<br>SAMPLE: small tissue/fragment	Push until breaking the fragment	claw	Grip	claw	Release	biobox
		Push until breaking the fragment	claw	Suction	suction sampler	Suction off	biobox
		Scrape	basket	Collect	basket	Hold	basket
Antipatharia	SHAPE: Arborescent<br>CONS&DEF: Rigid skeleton and wiry elastic tissue (that makes difficult to take fragments)<br>SAMPLE: whole tree	Grip the base and pull	claw	Hold	claw	Release	biobox<br>drawer
		Push until breaking the host	claw	Grip	claw	Release	biobox<br>drawer
Actinaria	SHAPE: Polyp, Big<br>CONS&DEF: soft to medium	Grip and pull	claw	Hold	claw	Release	biobox
	SHAPE: Polyp, Small<br>CONS&DEF: soft to medium	Scrape	suction sampler	Suction	suction sampler	Suction off	biobox
Zoantharia	SHAPE: Encrusting<br>CONS&DEF: soft to medium<br>SAMPLE: whole organism	See collection of its host (rocks, gorgonians, antipatharians)					
	SHAPE: Encrusting<br>CONS&DEF: soft to medium<br>SAMPLE: fragment	Scrape	suction sampler	Suction	suction sampler	Suction off	biobox
		Push until breaking the fragment/ the host	claw	Suction	suction sampler	Suction tube	jar

i.e.	Condition	Detach		Pick and place		Store	
Alcyonacea	DESIRED SAMPLE: Whole<br>SUBSTRATE: rigid<br>CONS&DEF: soft, elastic tissues, breakable skeleton	Push the base of the tree (close to the substrate) until breaking the base	claw	Grip	claw	Release	biobox<br>drawer
Alcyonacea	DESIRED SAMPLE: whole<br>SUBSTRATE: friable<br>CONS&DEF: soft, elastic tissues, breakable skeleton	Grip the base and pull	claw	Hold	claw	Release	biobox
Acanella<br>*Chrysogorgia*	DESIRED SAMPLE: whole<br>SUBSTRATE: rigid<br>CONS&DEF: rigid, brittle	Grip the base and pull	claw	Hold	claw	Release	biobox
Alcyonacea (gorgonians)<br>other Octocorallia spp.	DESIRED SAMPLE: fragment<br>SUBSTRATE: rigid<br>CONS&DEF: medium, Plastic (tissue tearing, skeleton) or brittle	Grip, twist and break	claw	Hold	claw	Release	biobox
		Grip, twist and break	claw	Suction	suction sampler	Suction off	biobox
Alcyonacea (gorgonians/sea whips)	DESIRED SAMPLE: whole<br>SUBSTRATE: rigid<br>CONS&DEF: soft to medium, Plastic (tissue tearing)	Grip and pull	claw	Suction	suction sampler	Suction off	biobox
Alcyonacea (gorgonians)	DESIRED SAMPLE: fragment<br>SUBSTRATE: rigid<br>CONS&DEF: medium, brittle<br>ANALYSIS: intact tissue, without abrasions	Scrape	basket	Collect	basket	Hold	basket
Pennatulacea	DESIRED SAMPLE: whole<br>SUBSTRATE: friable	Grip peduncule (not the leaves/polyps) and pull	claw	Hold	claw	Release	biobox
		Grip peduncule (not the leaves/polyps) and pull	claw	Suction	suction sampler	Suction off	biobox

i.e.	Condition	Detach		Pick and place		Store	
Ceriantharia	SAMPLE: whole	Insert jaws in the sediment, Grip and pull	claw	Suction	suction sampler	Suction	jar
Hydrocorals	SAMPLE: whole	Grip and pull	claw	Hold	claw	Release	biobox
Anthozoa	SAMPLE: with host, fragment	See host sampling (break the rock) or break a fragment					

Arthropods

i.e.	Condition	Detach		Pick and place		Store	
Chitonophilidae<br>Copepoda	DIMENSION: <1mm	See collection of its host (gastropods)					
Copepoda<br>Ostracoda<br>Amphipoda	DIMENSION: 0.1 : 1 cm	---	---	Suction	suction sampler	Suction off	biobox
Pycnogonida	DIMENSION: 2 : 3 cm<br>BEHAVIOR: crawler	Insert	corer	Pull	corer	Release corer	corer
Sessilia<br>Lepadiformes	DIMENSION: 1cm : 7 cm<br>SHAPE: Encrusting	Grip and pull	claw	Hold	claw	Release	biobox
Shrimps<br>Crabs<br>other Crustacea	DIMENSION: around 5-10 cm, occasionally bigger<br>LIFESTYLE: swimmer	---	---	Grip	claw	Release	biobox
		---	---	Suction	suction sampler	Suction tube	jar

Amphipoda<br>Decapoda (shrimps)<br>Ostracoda	DIMENSION: > 5 cm	---	---	Grip the trap	trap	Release the trap	trap

Mollusks

i.e.	Condition	Detach		Pick and place		Store	
Scaphopoda<br>Bivalvia<br>Gastopoda	CONS&DEF: Medium to Rigid<br>BEHAV: Buried, attached, crawling, respectively.	Scraping	claw (victor)<br>scoop	Scooping	claw (victor)<br>scoop	Release	biobox
		Scraping	claw<br>suction sampler	Suction	suction sampler	Suction tube	jar
		Grip carefully and pull	claw	Hold	claw	Release	biobox
Aplacophora	CONS&DEF: Soft	Scraping	claw (victor)<br>scoop	Scooping	claw (victor)<br>scoop	Release	biobox
		Scraping	claw<br>suction sampler	Suction	suction sampler	Suction tube	jar
Gastropoda	Parasitic	See collection of its host (gorgonians)					
Gastropoda<br>Aplacophora	ENV: on soft sediment	Insert	corer	Pull	corer	Release corer	corer
Cephalopoda	CONS&DEF: Soft	---	---	Grip	claw	Release	biobox
Bivalvia<br>Aplacophora		---	---	Grip the trap	trap	Release the trap	trap

Echinoderms

i.e.	Condition	Detach		Pick and place		Store	
Echinoidea	Rigid and brittle, with spines	Grip	claw (generic, victor, liropus)	Hold	claw (generic, victor, liropus)	Release	biobox
		Scoop, eventually with some environment	scoop	Hold	scoop	Pour	drawer
Asteroidea<br>Ophiuroidea	Medium	Grip	claw (generic, victor)	Hold	claw (generic, victor)	Release	biobox
		Scoop, eventually with some environment	scoop	Hold	scoop	Pour	drawer
Holothuroidea	Soft to medium	---	---	Grip	claw	Release	biobox
		---	---	Scoop, eventually with some environment	scoop	Pour	drawer
		---	---	Suction	suction sampler	Suction tube	jar
Crinoidea	Soft to medium<br>NEED: avoid that the thrusters or currents blow the sample away	Grip and pull	claw	Suction	suction sampler	Suction off	biobox

Polychaeta

i.e.	Condition	Detach		Pick and place		Store	
Polychaeta		---	---	Grip	claw	Release	biobox
	NEED: avoid that the thrusters or currents blows the sample away	Grip and pull	claw	Suction	suction sampler	Suction off	biobox
	ENV: on soft sediment	Insert	corer	Pull	corer	Release corer	corer
		---	---	Grip the trap	trap	Release the trap	trap

Porifera

i.e.	Condition	Detach		Pick and place		Store	
Porifera	ENV: breakable sediment<br>NEED: peduncule	Push to break or Grip and pull the rock	claw	Grip	claw	Release	biobox<br>drawer
	ENV: hard sediment	Grip the base and pull	claw	Hold	claw	Release	biobox
	ENV: hard sediment<br>NEED: avoid that the thrusters or currents blows the sample away	Grip and pull	claw	Suction	suction sampler	Suction off	biobox
	ENV: on soft sediment	Insert	corer	Pull	corer	Release corer	corer
	SHAPE Encrusting<br>SAMPLE Fragment/Tissue	Scraping	claw<br>suction sampler	Suction	suction sampler	Suction tube	jar
		Scraping	claw	Grip tissue	claw	Release	biobox
	SHAPE Encrusting	---	---	Grip rock	claw	Release	drawer

Other species

i.e.	Condition	Detach		Pick and place		Store	
Tunicata/ Ascidiacea	CONS&DEF: very soft to soft	Grip and pull	claw (generic, liropus)	Hold	claw	Release, or kept in the gripper because they would have floated out of the open basket available	biobox<br>claw
		Scoop	scoop	Hold	scoop	Pour	scoop
algae		Grip and pull	claw	Hold	claw	Release	biobox
		Grip the rock	claw	Hold	claw	Release	biobox

Hemichordata/ Enteropneusta		---	---	Grip the trap	trap	Release the trap	trap
Nemertea		Insert	corer	Pull	corer	Release corer	corer
Bryozoa	Custom tool, like a fork. SIZE: <1 mm	Scrape	custom rake	Hold	custom rake	Release	basket
Sipuncula		Insert	corer	Pull	corer	Release corer	corer
Thiolava veneris	Rock covered of white mat	Grip the rock	claw	Hold	claw	Release	biobox
marine snow	Dense water	?	?	?	?	?	?

Fluids and geological samples

i.e.	Condition	Detach		Pick and place		Store	
fluids		---	---	Put PEGAS near the outlet and close it	PEGAS	Release PEGAS	drawer
carbonate crust	Outcropping the sea bottom, in sites where there was gas flow exits	Lever: Insert the claw in a fissure and apply low bending moment to break the piece	claw	Grip	claw	Release	drawer
rock	Fragments of rocks already detached	---	---	Grip	claw	Release	drawer
sediment		Insert	corer	Pull	corer	Release corer	corer
		Insert Falcon tube in the sediment and move the robot	falcon	Lift Falcon tube	falcon	Release Falcon tube	falcon
