# Supplementary material for: Marine Robotics for Deep-Sea Specimen Collection: A Taxonomy of Underwater Manipulative Actions
Source: Sensors (Basel). 2022 Feb 14;22(4):1471. doi: 10.3390/s22041471 (PMC8878465; doi:10.3390/s22041471)
Supplement: Supplementary file 1 [file sensors-22-01471-s001.zip › sensors-1539539-supplementary/Annex_D_Researchers_comments_v06.pdf]

Annex D

Common sampling gears and ideal features needed for sampling: Researchers' comments

D. Freetext comments from researchers

D.1. Comments on gears commonly used during sampling procedures

Here, we report some interesting issues the researchers reported about the gears commonly used in the sampling procedures, in particular about the *grippers*, the *suction sampler* and the *storage containers*.

Here follows what researchers reported related to the *grippers*:

- Claws are suitable for large epibenthic animals, but not suitable for the very small animals that can exit the gripper (i.e. from the groove of the T-handle, or being smaller than the maximum closure range of the gripper).
- The closure range is an important parameter: full closure of the claw might destroy the sample, while limited closure range makes very small animals impossible to pick.
- Claws most of the times do not give feedback to the pilot, and the closure level is commanded by the experience of the pilot. Grasping force is difficult to be tuned, and this makes the claw not the perfect instrument for soft bodied animals.
- The speed of the closure is significant: fast closing is useful for swimming animals (even if often they swim too fast to be followed with the manipulator) or for organisms that retracts (i.e. anemones, pennatulaceans); moreover, very strong currents might influence the closure of the claw, producing vibrations.
- Once a sample is collected, it's difficult to prevent unwanted release during drag, or caused by currents or thrusters flow.

Here follows what researchers reported related to the *suction sampler*:

- The dimensions of the tube of the sampler are critical: if it gets clogged, it cannot be used for the rest of the campaign. The narrower rigid tip of the sampler serves as a reference for the allowed dimensions.
- The consistency of the sample is taken into account when the suction sampler is used, because a rigid sampler is more likely to clog the tube, even if it's small, but a very soft sample would tear apart in the tube, or by repeatedly turning in the jar.
- The suction power cannot be tuned, but it could be too low to detach the sample, or too strong that the sample is ruined because it is scraped when passing through the tube or turning into the jar.
- Suction sampler is useful for small swimming crustaceans or mollusks, but only when paired with a manipulator that has good workspace, because positioning the sampler very close to the sample is crucial.
- Suction flow might alter some kind of analysis (i.e. estimation of number of microorganism hosted on the sample).

Here follows what researchers reported related to the *storage systems*:

- The insertion of the samples in the containers is not an easy operation and it is poorly automatized, because ROVs were not thought for sampling.
- It's important to check for the accessibility of the container during the pre-dive.
- The dimensions of the container are decided during the pre-dive, and it might not be suitable for the samples found during the exploration. Problems are encountered especially with big tubeworms or rocks.
- Pooring in a biobox ability might be useful.

Mazzeo, A.; Aguzzi, J.; Calisti, M.; Canese S.; Angiolillo M.; Allcock A.L.; Vecchi F., Stefanni, S.; Controzzi, M.; Marine Robotics for Deep-sea Specimen Collection: A Taxonomy of Underwater Manipulative Actions. *Sensors*.

Publisher's Note: MDPI stays neutral with regard to jurisdictional claims in published maps and institutional affiliations.

Copyright: © 2021 by the authors. Submitted for possible open access publication under the terms and conditions of the Creative Commons Attribution (CC BY) license (http://creativecommons.org/licenses/by/4.0/).

- Buoyancy, currents or ROV movements limit the use of multiple racks within the same box, because when reopening a drawer the samples might flew out: sometimes a stone is put on the samples to ensure it does not float out.
- The position of the storage container is important especially for ROVs that does not implement a drawer (i.e. if the collecting basket is below, it's difficult to move near to the sea bottom; if it's above it's more difficult to insert it).

D.2. Comments on desired features of the ideal tool

Here, we report some interesting features the researchers reported as ideal in the design of a new tool for the sampling procedures.

- A claw is useful for detachment purposes: it does not fully close not to destroy the sample, but it would be useful to also extend its tips to tweezers-like endings to take very small samples.
- Wrapping the sample in a 500 μm sieve would avoid the loss of the microorganisms that are attached to a sample, and the loss of sample itself during drag.
- A rigid exposed finger would be useful for scrap.
- A hollow and rigid part is useful for scooping or scraping, or scraping+collecting.
- Two soft parts enveloping the sample are useful to hold it without squashing.
- When the suction is used, it would be useful to tune suction power.
- When the corer is used, a function that brings the corer up and back to place vertically is useful, in order not to disturb your sample stratification (for example, Victor has this control).
- For corals, a cutting tool like a pruning shear would be useful, ideally cutting a piece that is simultaneously hold by the claws.
- A general-purpose tool would be ideal, as changing tool requires time.
